# Characteristics and Functions of Dominant Yeasts Together with Their Applications during Strong-Flavor Baijiu Brewing

**DOI:** 10.3390/foods13152409

**Published:** 2024-07-30

**Authors:** Weiwei Dong, Yulun Zeng, Jiyuan Ma, Kaiyun Cai, Tingting Guo, Guangxun Tan, Xiang Yu, Yuanliang Hu, Nan Peng, Shumiao Zhao

**Affiliations:** 1 Hubei Key Laboratory of Edible Wild Plants Conservation and Utilization, College of Life Sciences, Hubei Normal University, Huangshi 435002, China; weiweidong@hbnu.edu.cn (W.D.); 19546877618@163.com (J.M.); ylhu@hbnu.edu.cn (Y.H.); 2 National Key Laboratory of Agricultural Microbiology and College of Life Science and Technology, Huazhong Agricultural University, Wuhan 430070, China; 3 Hubei Daohuaxiang Liquor Co., Ltd., Yichang 443112, China

**Keywords:** strong-flavor baijiu, yeast, reinforced *Fuqu*, microbiota, flavor substances

## Abstract

Yeasts are pivotal brewing microbes that are associated with the flavor and quality of Chinese baijiu, yet research on dominant yeasts in strong-flavor baijiu brewing remains limited. In this study, *Saccharomyces cerevisiae*, *Pichia kudriavzevii*, and *Kazachstania bulderi* were identified as predominated yeasts in strong-flavor baijiu. Each strain showed distinct characteristics in ethanol resistance, thermal tolerance, and lactic acid tolerance, severally. *S. cerevisiae* FJ1-2 excelled in ethanol and ethyl ester production, *P. kudriavzevii* FJ1-1 in ethyl acetate, and *K. bulderi* FJ1-3 in lactic acid generation. Subsequently, the reinforced *Fuqu* of each yeast were severally prepared for application in baijiu brewing to verify their functions. Results revealed that the relative abundance of fortified yeast in each group rose. *Pichia*, *Kazachstania*, and *Saccharomyces* emerged as the core microbe for each group, respectively, by co-occurrence network analysis, influencing the microbiota to regulate flavor substances. In short, *P. kudriavzevii* FJ1-1 enhanced ethyl acetate. *K. bulderi* FJ1-3 improved ethyl caproate production and decreased levels of ethyl acetate and higher alcohols by modulating yeast community between *Pichia* and *Saccharomyces*. This is a systematic endeavor to study the functions of yeasts of strong-flavor baijiu, providing a solid basis for improving baijiu quality.

## 1. Introduction

**Figure 1:**
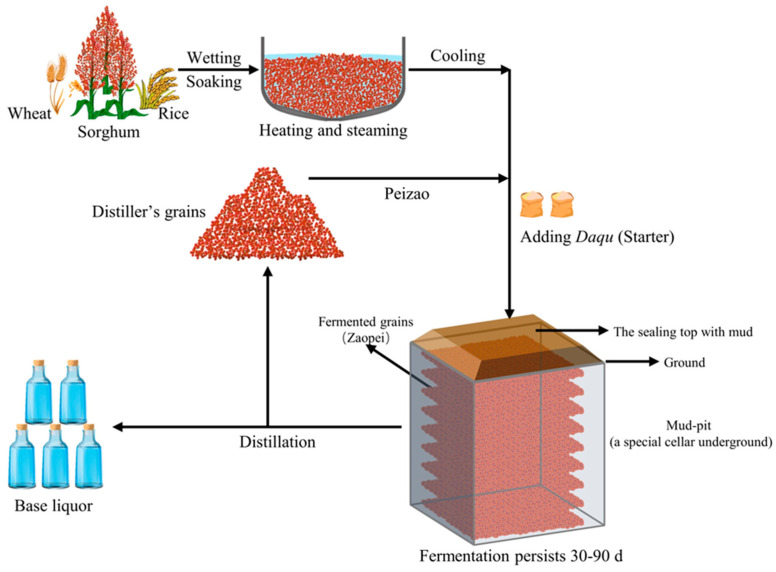
Schematic diagram of strong-flavor baijiu brewing.

Baijiu, one of the six most commonly distilled spirits in the world, is a distinctive and valuable alcoholic beverage in China. According to data from the National Statistics Bureau, in 2022, the production of baijiu reached 6.71 billion liters, with sales soaring to 85.10 billion euros and achieving a profit of 28.28 billion euros [1], making a significant contribution to the steady growth of the national economy. Baijiu is categorized into four primary types based on flavor: strong-flavor, sauce-flavor, light-flavor, and rice-flavor. Among these, strong-flavor baijiu enjoys widespread recognition and preference by Chinese consumers, commanding over 60% of the market share of baijiu in China, playing a pivotal role in the development of the baijiu industry [2,3]. Although the brewing techniques of strong-flavor baijiu vary by region, their key processes remain largely consistent (Figure 1). These processes include grains (mainly sorghum) crushing, soaking, distilling with previously fermented grains from the preceding brewing cycle, mixing thoroughly with *Daqu* powder after spreading and cooling, sealing, and fermenting in mud-pit for 60–90 days. Subsequently, the fermented grains are spaded out and distilled to obtain base liquor, stored, and then blended to produce finished baijiu for sale [3,4,5,6].

Throughout the brewing processes of strong-flavor baijiu, a diverse array of microbes settles on the fermented grains, where they grow, proliferate, and metabolize. Thus, microbes constitute the cornerstone and essence of baijiu fermentation. Dominant fungi in strong-flavor baijiu include *Kazachstania*, *Saccharomyces*, *Pichia*, *Issatchenkia*, *Candida*, *Aspergillus*, *Thermoascus*, and *Thermoyces* [5,7,8]. *Lactobacillus* predominates among the bacteria, comprising 65% to 95% of the microbial abundance, while other prevalent bacteria encompass *Acetobacter*, *Bacillus*, *Streptomyces*, and *Streptococcus* [5,7,8]. Current research on pit mud microbes primarily centers on caproic acid bacteria, with *Clostridium* and *Caproicibacterium* as dominant genera [5,9,10]. Within the above brewing microbes, molds, *Thermoascus*, *Thermoyces*, and *Bacillus* mainly appeared in the early and middle stages of fermentation, degrading starch into reducing sugars and breaking down proteins into peptides or amino acids to facilitate the growth of lactic acid bacteria and yeasts. Lactic acid bacteria and yeasts dominated throughout fermentation, rapidly proliferating and generating substantial quantities of organic acids (lactic acid and acetic acid) and ethanol as metabolic products [11]. Simultaneously, these produced reducing sugars, ethanol, and some organic acids served as carbon and energy sources to promote the growth of caproic acid bacteria and caproic acid synthesis [2,8,12]. Moreover, dominant microbes, such as yeasts, molds, and lactic acid bacteria, possessed esterase activity, catalyzing esterification reactions between ethanol and organic acids to produce esters [11,13]. Here, ethyl caproate, ethyl butyrate, ethyl lactate, and ethyl acetate constituted the four major esters closely related to the quality of strong-flavor baijiu [2,14]. Therefore, the brewing of strong-flavor baijiu entails the cooperative fermentation of multiple microbes. Despite that, yeasts were the most important microbes that spanned the entire brewing cycle, producing the majority of ethanol and expressing esterases involved in the synthesis of four major esters during brewing [14,15,16].

Regarding the yeast community in fermented grains during the brewing of strong-flavor baijiu, it mainly includes *Kazachstania*, *Saccharomyces*, *Pichia*, *Issatchenkia*, *Candida*, *Geotrichum*, *Debaryomyces*, *Naumovozyma*, and *Trichosporon* [5,7,8,17,18]. You et al. isolated *Kazachstania exigua*, *Geotrichum silvicola*, *Pichia kudriavzevii*, *Saccharomyces cerevisiae*, *Zyosaccharomyces bailii*, and *Kazachstania humilis* from the fermented grains of strong-flavor baijiu by using pure culture techniques [18]. Li et al. identified *Candida*, *Debaryomyces*, and *Pichia* as dominant yeast genera in fermented grains of strong-flavor baijiu using denatured gradient gel electrophoresis technology [17], while Liu et al. determined *Saccharomyces*, *Kazachstania*, *Naumovozyma*, and *Trichosporon* as dominant yeast genera by using amplicon sequencing, noting variations in their community structures at different stages [3]. Hu et al. revealed that *Saccharomyces* and *Pichia* were the main yeast genera involved in ethanol production during the brewing of strong-flavor baijiu through RNA sequencing, with *Saccharomyces* being the most significant contributor [16].

Although the diversity of yeasts has been studied during the brewing of strong-flavor baijiu, the characteristics and functions of these yeasts are not fully understood. Therefore, this study focused on the isolating of dominant yeast strains from the brewing of strong-flavor baijiu to investigate their characteristics and functions. Specifically, fermented grains were collected to study yeast community and to isolate dominant yeasts, whose characteristics and functions were then clarified. This research aims to elucidate the functions of yeasts, offering potential yeast resources for enhancing the quality of strong-flavor baijiu in the future.

## 2. Materials and Methods

### 2.1. Analysis of Yeast Community, Count, Isolation, and Identification

The fermented grains were sampled in a strong-flavor baijiu brewing factory located in the middle reaches of Yangtze River (Latitude 30.72° N, Longitude 111.49° E). Here, we collected fermented grains on 0 d, 5 d, 10 d, 30 d, 60 d, and 90 d during fermentation. After sampling, the fermented grains were stored in an ice box, and then immediately transferred to lab for analysis. To analyze the yeast community, the amplicon sequencing based on Internal Transcribed Spacer (ITS) region was performed [3]. Meanwhile, the enumeration of dominant yeasts, traditional dilution and plating method was used according to our previous study with little modification [19]. Specifically, 10 g fermented grains of each sample were added to 90 mL of sterile normal saline and shaken at 30 °C and 200 rpm for 20 min. Afterward, supernatants were piped up for gradient dilution and plated on Rose Bengal (RB) agar and Wallerstein Laboratory (WL) nutrient agar with 100 μg/mL of ampicillin. Following incubation at 30 °C for 2 days, we enumerated yeasts based on their colony numbers on the plates, and then identified them on the strength of colony morphologies, colony PCR (primer pair NL1 and NL4), and Sanger sequencing (Supplementary Material).

### 2.2. Characterizations and Metabolic Profiles of Dominant Yeasts

The characteristics of dominant yeasts were evaluated using Yeast Peptone Dextrose (YPD, Solarbio) medium. Temperature tolerance was determined by incubating yeasts at different temperatures (25, 30, 37, 40, and 45 °C) under static culture for 24 h. For other tolerance of yeasts, YPD medium with different content of ethanol (0, 2%, 4%, 6%, 8%, 10%, and 12%), and lactic acid (0, 1%, 2%, 4%, 6%, 8%, and 10%) was applied separately, and then statically incubated at 30 °C for 24 h. Afterward, the cell density at 600 nm was detected.

To detect the metabolic profiles of these yeasts, sorghum juice medium (Supplementary Materials) was prepared and used to simulate baijiu brewing under the static condition. Each yeast was incubated at 30 °C for 5 days with initial cell density at 1 × 10^6^ CFU/mL, severally. After incubation, supernatants of the culture were collected for metabolites analysis by high-performance liquid chromatography (HPLC) and head-space solid-phase microextraction and gas chromatography–mass spectrometry (HS-SPME-GC–MS) [19,20].

### 2.3. Preparation of Reinforced Fuqu

A single colony of each yeast strain was inoculated into 15-mL YPD medium, respectively. After incubation at 30 °C and 200 rpm for 20 h, the second inoculation was completed with a ratio of 5% into 200 mL of YPD medium. Simultaneously, the wheat bran medium was prepared by autoclaving 40 g of wheat bran in a 500-mL canning jar at 121 °C for 30 min. Further, the YPD culture was all inoculated into wheat bran medium at a ratio of 25% and incubated at 30 °C for 2 days via solid-state fermentation. Stirring was carried out every 6 h to increase the oxygen content of fermentation. At last, the cultured wheat bran medium was dried at 37 °C for 12 h, and the number of yeast cells was counted via the above dilution and plating method [19]. Hence, three kinds of reinforced *Fuqu*, containing *S. cerevisiae* FJ1-2 *Fuqu* (SF), *P. kudriavzevii* FJ1-1 *Fuqu* (PF), and *K. bulderi* FJ1-3 *Fuqu* (KF), were made for further baijiu brewing at lab scale.

### 2.4. Functional Verification of Dominant Yeasts via Reinforced Fuqu Application

The well-prepared brewing grains before adding *Daqu* in the baijiu producing workshop were used as materials. Thereinto, three experiment groups (SF, PF, and KF) and one control check (CK) group were set to verify the functions of these three dominant yeasts (Table 1). For CK group, 2 kg of *Daqu* and 20 kg of brewing grains were fully mixed, put into a sterile plastic case, sealed, and transferred to the lab for spontaneous fermentation with a period of 45 days. For each reinforced *Fuqu* group, 10% of *Daqu* was replaced by one kind of *Fuqu*, and other conditions were same to CK group. Samples of fermented grains were collected on days 1, 5, 15, 30, and 45 for subsequent analyses.

The physicochemical factors (moisture, pH, acidity, and reducing sugar) of fermented grains were analyzed according to our previous studies [3,21]. Flavor substances in fermented grains were extracted by taking 5 g of sample suspended in 20 mL of ddH_2_O with ultrasonication at 0 °C for 30 min, as well as centrifugation at 4000× *g* for 10 min at 4 °C. Adjacently, the supernatant was filtered through 0.22-μm filters for above HPLC and HS-SPME-GC-MS analyses (19). The microbial community of fermented grains at different times within four groups was evaluated, via amplicon sequencing based on the V3-V4 region of 16S rDNA and near full-length ITS. After the total DNA of fermented grains was extracted, the bacterial and fungal libraries were constructed through PCR with primer pair 338F (ACTCCTACGGGAGGCAGCA) and 806R (GGACTACHVGGGTWTCTAAT), in cooperation with ITS1F (CTTGGTCATTTAGAGGAAGTAA) and LR3 (CCGTGTTTCAAGACGGG), respectively. Furthermore, the libraries were severally sequenced by Illumina MiSeq platform and PacBio Sequel II platform, respectively.

### 2.5. Bioinformatics Analysis and Data Availability

The bioinformatic analyses of this study were carried out with QIIME 2 (2019.4) according to the detailed procedures in our previous study with the help of Personalbio platform [19]. Raw sequences generated by sequencing in the present study have been deposited to National Center for Biotechnology Information (NCBI) with Bio Project accession number PRJNA872358 for the bacterial community, and PRJNA872371 for the fungal community.

### 2.6. Statistical Analysis

All data were processed by Excel (version 2021). One-Way ANOVA was performed to evaluate the significant differences (*p* < 0.05). The same letter represented no significant differences between the chosen groups, while different letters revealed significant differences between the chosen groups.

## 3. Results

### 3.1. Yeast Community, Count, Isolation, and Identification

**Figure 2:**
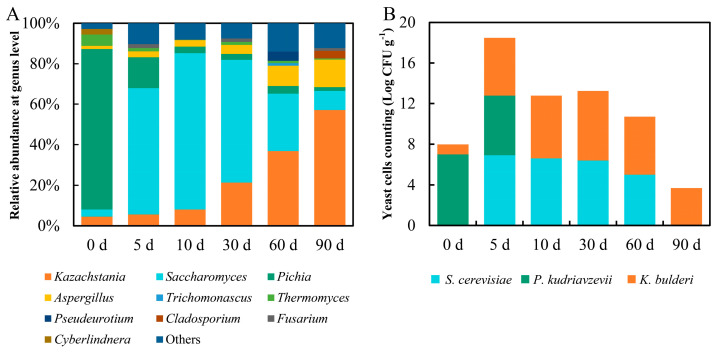
The composition of fungal community (**A**) and the yeast cells counting (Log CFU g^−1^) of dominant yeasts (**B**) in fermented grains during strong-flavor baijiu brewing.

The fungal community during brewing processes of strong-flavor baijiu was analyzed based on amplicon sequencing directing at ITS region of rDNA. Results revealed that *Kazachstania* (4.52–57.10%), *Saccharomyces* (3.50–78.01%), *Pichia* (1.82–79.22%), and *Trichomonascus* (0–1.26%) constituted the yeast community (Figure 2A). Here, *Kazachstania*, *Saccharomyces* and *Pichia* were the dominant yeasts with relative abundances always over 1%, while *Trichomonascus* only showed up on day 60 with relative abundance over 1% (Figure 2A). Coincidentally, the results from traditional dilution and plating unveiled that there were three types of yeasts on all plates based on colony and cell morphologies. Sanger sequencing identified three species, including *S. cerevisiae*, *P. kudriavzevii*, and *K. bulderi* (Figure S1). Thus, the dominant yeasts of this strong-flavor baijiu belonged to *S. cerevisiae*, *P. kudriavzevii*, and *K. bulderi*. Meanwhile, *P. kudriavzevii* and *K. bulderi* coexisted on day 0, followed by the showing up of *S. cerevisiae* on day 5. *P. kudriavzevii* was entirely superseded by *K. bulderi* and *S. cerevisiae* by day 10, and they collaborated until day 60. At last, only *K. bulderi* was present on day 90 (Figure 2B). In terms of yeast numbers, *P. kudriavzevii* had the highest cell count of 10^6.9^ CFU/g on day 0, while *S. cerevisiae* reached the same level on day 5 (Figure 2B). Regarding *K. bulderi*, it exhibited a trend of increase first followed by decline. The number of *K. bulderi* reached 10^6.8^ CFU/g on day 30 (Figure 2B). Importantly, the number of these three yeasts species remained over 10^5^ CFU/g at most time of the brewing cycle, hinting that this number of yeasts could maintain their functions well on ethanol production and flavor substances formation during strong-flavor baijiu brewing [18].

### 3.2. Characterizations of Dominant Yeasts

**Figure 3:**
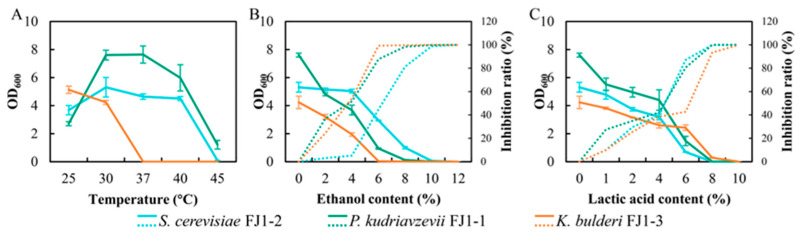
The characteristics of isolated yeasts on temperature tolerance (**A**), ethanol tolerance (**B**), and lactic acid tolerance (**C**). Here, the continuous line represents the OD600, while the broken line states the inhibition ratio.

After identification, three strains from dominant yeasts encompassing *S. cerevisiae* FJ1-2, *P. kudriavzevii* FJ1-1, and *K. bulderi* FJ1-3 were used for further study. *P. kudriavzevii* FJ1-1 showed the best thermal tolerance, proliferating at 45 °C, while *S. cerevisiae* FJ1-2 displayed similar thermal tolerance to *P. kudriavzevii* FJ1-1, but its cell density was lower than that of *P. kudriavzevii* FJ1-1 (Figure 3A). *K. bulderi* FJ1-3 preferred a low temperature of ≤30 °C, and its thermal tolerance was not good, even being unable to survive at 37 °C (Figure 3A). For ethanol tolerance, *S. cerevisiae* FJ1-2 gained the highest resistance to ethanol, with its growth barely affected by an ethanol content of less than 4% (Figure 3B). However, a significant inhibition by ethanol was observed for the other two yeasts, and the inhibition ratio of *K. bulderi* FJ1-3 was lower than that of *P. kudriavzevii* FJ1-1 when ethanol content was less than 4% (Figure 3B), suggesting that *K. bulderi* FJ1-3 gained higher survival ability over *P. kudriavzevii* FJ1-1 under this range of ethanol content. For lactic acid tolerance, *K. bulderi* FJ1-3 exhibited the lowest inhibition ratio among these three yeasts (Figure 3C), intimating that *K. bulderi* FJ1-3 possessed the best tolerance to lactic acid. Usually, the brewing environment of strong-flavor baijiu tends to be high ethanol and lactic acid, which may drive the above yeast succession.

### 3.3. Metabolic Profiles of Dominant Yeasts

**Figure 4:**
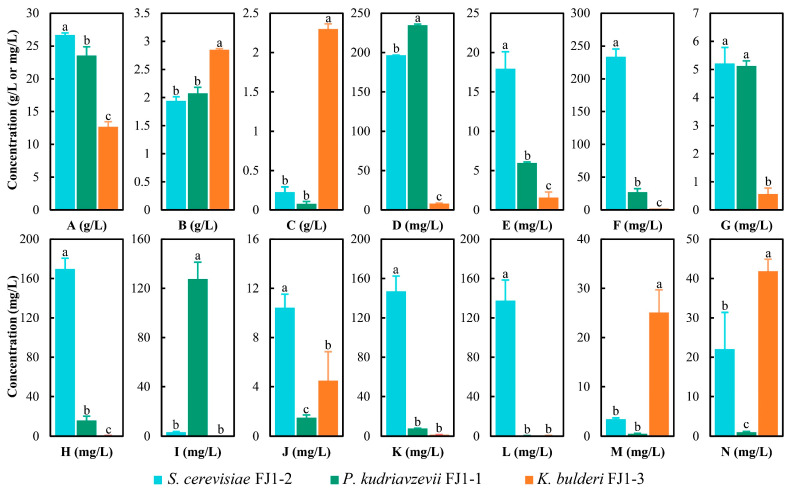
The metabolites of the three yeasts cultured by sorghum juice medium, including ethanol (**A**), lactic acid (**B**), propionic acid (**C**), acetic acid (**D**), caproic acid (**E**), octanoic acid (**F**), nonanoic acid (**G**), decanoic acid (**H**), ethyl acetate (**I**), ethyl caproate (**J**), ethyl octoate (**K**), ethyl caprate (**L**), ethyl benzoate (**M**), and phenol (**N**).

Although the characteristics of yeasts was uncovered, their metabolic profiles during fermentation were not clear. Hereof, the metabolic profiles of these yeasts revealed that ethanol was the main metabolite, whose content reached 26.69 g/L, 23.56 g/L, and 12.68 g/L for *S. cerevisiae* FJ1-2, *P. kudriavzevii* FJ1-1, and *K. bulderi* FJ1-3, respectively (Figure 4A). The ethanol-producing aptitude of *S. cerevisiae* FJ1-2 was the best (Figure 4A), which corresponded to the results of the ethanol tolerance (Figure 3B). With respect to other metabolites, they produced organic acids, ethyl esters, and phenol (Figure 4). Lactic acid and propionic acid were the dominant organic acids (Figure 4B,C). *K. bulderi* FJ1-3 yielded significantly higher lactic acid than the other two yeasts (Figure 4B), which was consistent with the results of lactic acid tolerance (Figure 3C). Interestingly, *K. bulderi* FJ1-3 also seized a significantly higher ability to generate propionic acid than the other two yeasts (Figure 4C). *P. kudriavzevii* FJ1-1 gained significantly higher acetic acid and ethyl acetate content after fermentation (Figure 4D,I). *S. cerevisiae* FJ1-2 produced more medium-chain fatty acids (MCFAs) (Figure 4E–H) and their ethyl esters (Figure 4J–L). Moreover, *K. bulderi* FJ1-3 maintained a higher ability to generate benzene-related chemicals including ethyl benzoate and phenol (Figure 4M,N). These three yeasts exhibited different metabolic profiles during fermentation, thus cooperating with each other to influence flavor substances of baijiu brewing.

### 3.4. Dynamics of Physicochemical Factors during Reinforced Fuqu Application

**Figure 5:**
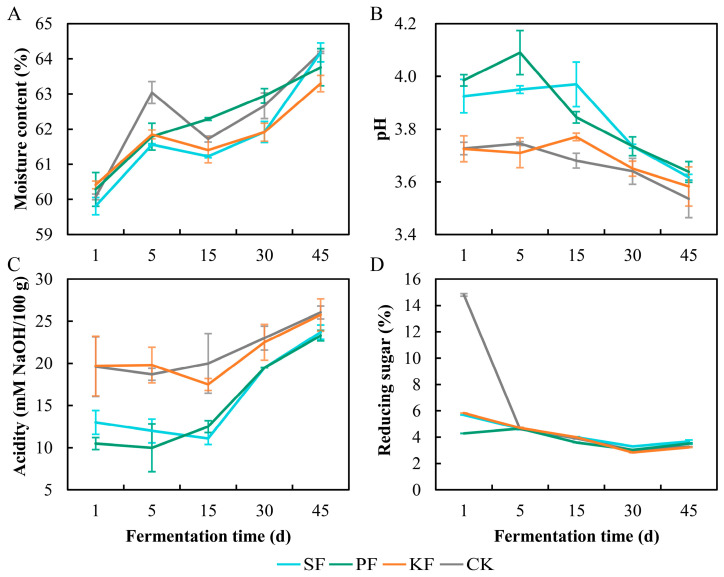
Physicochemical factors of fermented grains during the application of reinforced Fuqu in baijiu brewing at lab scale, includes moisture (**A**), pH (**B**), acidity (**C**), and reducing sugar (**D**).

The moisture of the four groups showed an increasing trend (Figure 5A), and this uptrend could be attributed to microbial activity that metabolizes macromolecules and produces H_2_O [18]. However, the pH and acidity exhibited opposite dynamics among the groups, with SF and PF grouping together and differing from KF and CK (Figure 5B,C). In this regard, KF and CK groups had higher acidities than those of SF and PF groups during the whole fermentation cycle (Figure 5C), outlining that more organic acids were produced. As for the change in reducing sugar, a broadly similar trend was observed: it dropped from day 1 to day 30 and then stabilized (Figure 5D). However, significant differences in content of reducing sugar were observed among the four groups on day 1 (Figure 5D), with the CK group exhibiting a significantly higher content than the other three groups (Figure 5D). This suggests that the additional yeast cells introduced from reinforced *Fuqu* consumed a large amount of reducing sugar within one day. In addition, *P. kudriavzevii* possessed better reducing sugar metabolic capability than the other two yeasts since the SF group displayed the lowest reducing sugar content on day 1 (Figure 5D). This phenomenon supported that *P. kudriavzevii* occupied the most proportion of yeast community at the beginning of fermentation in situ (Figure 2), as it could quickly consume reducing sugar for growth and propagation and might capture more ecological niches [22].

### 3.5. Dynamics of Flavor Substances during Reinforced Fuqu Application

**Figure 6:**
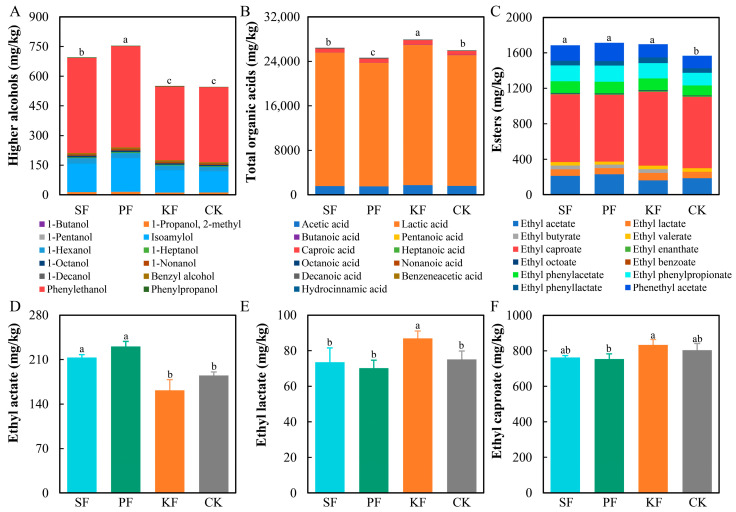
Three main flavor substances of fermented grains during the application of reinforced Fuqu in baijiu brewing at lab scale, including higher alcohols at top 12 (**A**), total acids at top 11 (**B**), total esters at top 12 (**C**), ethyl acetate (**D**), ethyl lactate (**E**), and ethyl caproate (**F**).

During baijiu brewing, microbes played vital roles in producing ethanol and many other metabolites comprising higher alcohols, organic acids, esters, and other compounds [23,24]. Here, a total of 140 kinds of chemicals were detected, with the main alcohols, organic acids, and esters shown in Figure S2. Ethanol, lactic acid, and acetic acid were the most abundant three metabolites during fermentation regardless of group settings. The ethanol content presented a rising tendency (Figure S2A), whose final concentration ranged from 42.16 g/kg to 44.16 g/kg. As for higher alcohols, isoamylol and phenyl-ethanol were predominant, with declining trends (Figure S2B,C). For organic acids, acetic acid, lactic acid, and caproic acid dominated the whole fermentation with fluctuating, and their final concentration of KF group was all the highest (Figure S2D–F). When considering the ethyl esters, ethyl acetate (Figure S2G), ethyl lactate (Figure S2H), ethyl butyrate (Figure S2I), ethyl caproate (Figure S2J), ethyl phenylpropionate (Figure S2K), and ethyl phenylacetate (Figure S2L) were the main six types accounting for over 80% of the total esters. Thereinto, ethyl caproate and ethyl acetate were the top two esters, with ethyl caproate content rising and ethyl acetate content dropping during fermentation (Figure S2G,J). Furthermore, the final concentration of higher alcohols, total organic acids, and total esters was calculated (Figure 6). The results showed KF and CK groups gained lower higher alcohols content, but the organic acids content of KF group was the highest (Figure 6A,B). Additionally, the final content of total esters showed no significant difference among three reinforced *Fuqu* groups, but higher than that in CK group (Figure 6C). Meanwhile, reinforced *Fuqu* affected the compositions of esters, particularly the final content of ethyl caproate, ethyl lactate, and ethyl acetate (Figure 6D–F). The final content of ethyl caproate in KF and CK groups was higher than that of SF and PF (Figure 6D), accompanied by lower final content of ethyl acetate in KF and CK groups (Figure 6E). Overall, we could conclude that the reinforced *Fuqu* had a significant impact on the compositions of flavor substances during baijiu brewing at lab scale, especially the higher alcohols, organic acids, and esters, thus affecting the quality of baijiu.

### 3.6. Microbial Community during Reinforced Fuqu Application

**Figure 7:**
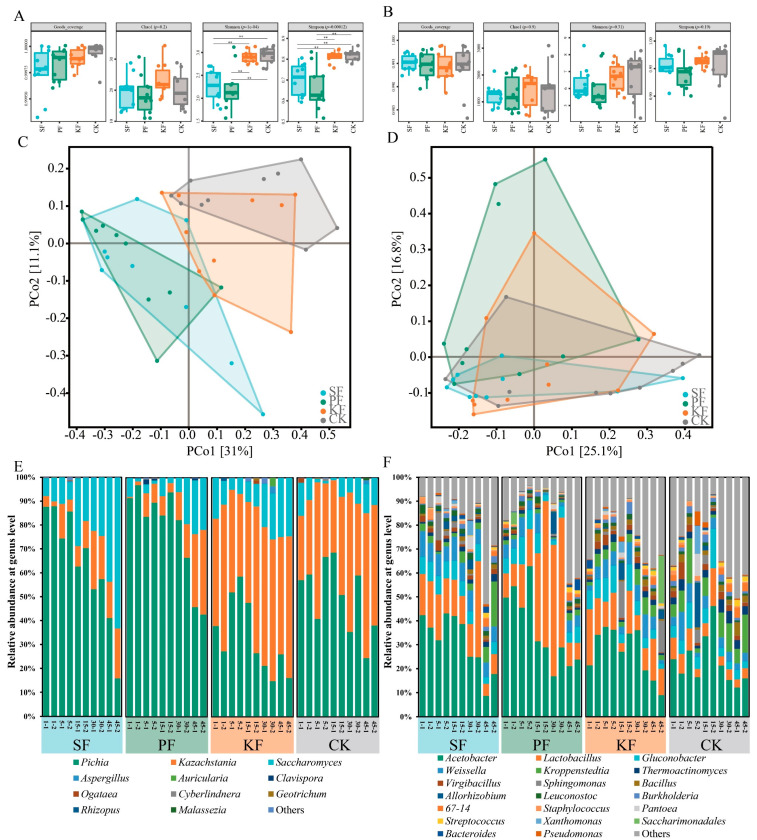
Microbiota in fermented grains during baijiu brewing at lab scale with the application of reinforced *Fuqu*. Here, fungal community α-diversity (**A**), bacterial community α-diversity (**B**), fungal community β-diversity (**C**), bacterial community β-diversity (**D**), fungal community composition (**E**), and bacterial community composition (**F**) (BrayCurtis distance (R2 = 0.8384, *p* = 0.0001) was used for β-diversity with confidence at 0.95).

After statistics, 3 phyla, 21 genera, and 29 species from the fungal community were identified, while 43 phyla, 1004 genera, and 1069 species from the bacterial community were found. The microbial diversity of each group that applied reinforced *Fuqu* was then calculated. Here, the goods coverage of sequences in each group was higher than 0.99 regardless of fungi and bacteria (Figure 7A,B), representing that the data of amplicon sequencing was of adequate depth and authenticity. For fungal α-diversity, the KF and CK groups gained higher values than those of SF and PF groups at Shannon and Simpson indexes, expect Chao 1 richness (Figure 7A). Similar results were found at bacterial α-diversity in these four groups (Figure 7B). For fungal β-diversity, groups SF and PF had similar community structures, while groups KF and CK had similar structures to some extent by principal component analysis (PCoA) (Figure 7C). However, bacterial β-diversity results were complicated, and all four groups shared some areas (Figure 7D), indicating that their bacterial community structures differed slightly.

After determining the diversity of microbial community, the microbial compositions and dynamics within these four groups were investigated. For fungal community, *Saccharomyces*, *Pichia*, and *Kazachstania* monopolized the whole fermentation (Figure 7E). The top five bacterial genera belonged to *Acetobacter*, *Lactobacillus*, *Gluconobacter*, *Weissella*, and *Kroppenstedtia* (Figure 7F). Here, attention was paid to the yeast community, since the yeast was strengthened during baijiu brewing. In CK group, *Saccharomyces* and *Kazachstania* increased in abundance during fermentation while *Pichia* decreased (Figure 7E). In SF group, *Saccharomyces* and *Kazachstania* gradually replaced *Pichia*, and the relative abundance of *Saccharomyces* increased to 43.6–63.1% on day 45 (Figure 7E). However, the relative abundances of *Kazachstania* and *Pichia* was reduced and promoted, respectively, compared with CK group (Figure 7E). In PF group, although *Pichia* was gradually superseded by *Saccharomyces* and *Kazachstania*, the relative abundance of *Pichia* peaked on day 1 and always kept at higher levels (96.9–42.7%) compared with CK group (Figure 7E). In KF group, the relative abundance of *Kazachstania* ranged from 30.1% to 75.1%, and the abundance of *Pichia* was reduced obviously in comparison with SF, PF, and CK groups, while the abundance of *Saccharomyces* was promoted compared with PF and CK groups (Figure 7E). Hence, the usage of reinforced *Fuqu* prepared from dominant yeast worked well, as the related yeast in each reinforced *Fuqu* group exhibited higher relative abundance than the CK group, affecting the microbiota during baijiu brewing (Figure S3). In short, fortifying *S. cerevisiae* FJ1-2 or *P. kudriavzevii* FJ1-1 would alter the microbiota by being beneficial to *Pichia*, thus reducing the abundance of *Kazachstania*. Strengthening *K. bulderi* FJ1-3 mainly reduced the abundance of *Pichia* and enriched that of *Kazachstania*.

### 3.7. Functions Verification of Dominant Yeasts and Their Relationships to Flavor Substances

**Figure 8:**
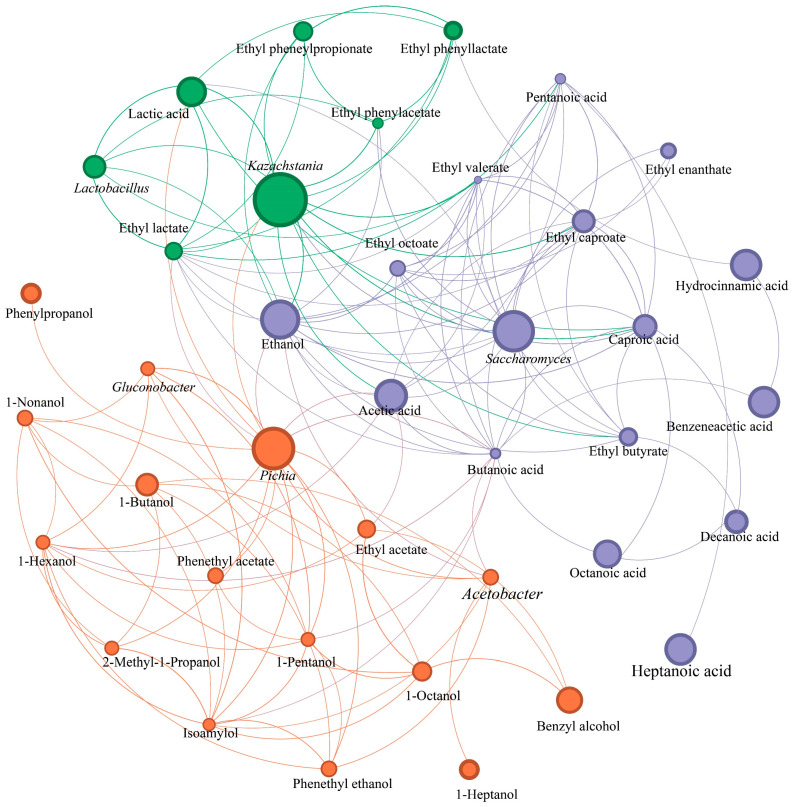
Co-occurrence network analysis of the dominant microbial genera and flavor compounds (similarity threshold = 0.6, *p* < 0.05).

**Figure 9:**
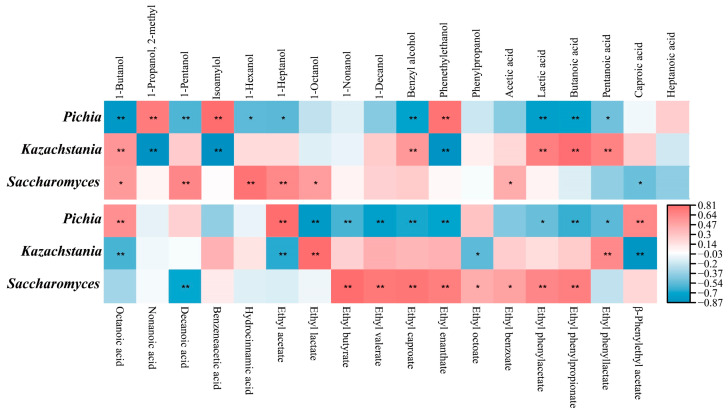
Spearman heatmap of relationships between core yeasts and flavor compounds (* *p* < 0.05, and ** *p* < 0.01).

To clarify whether the added fortified yeast was the core microbe for each group to function, the co-occurrence network analysis of the dominant microbial genera and flavor substances was carried out. Results showed that this network included three modules (Figure 8). *Pichia*, *Kazachstania*, and *Saccharomyces* emerged as the core microbes for each module, respectively (Figure 8). The interactions between these three yeasts existed and closely correlated with flavor formation (Figure 8), proving that yeast interactions were beneficial to ethanol fermentation and flavor formation [5,25,26]. Here, all three kinds of yeast were closely linked to ethanol production (Figure 8). *Pichia* exhibited a significant association with ethyl acetate, *Kazachstania* demonstrated a tight relationship with ethyl lactate, while *Saccharomyces* showed a strong connection with MCFAs and their ethyl esters (Figure 8). Subsequently, the concrete relationships between yeasts and flavor substances during baijiu brewing were clarified by the Spearman correlation between core yeasts and flavor compounds. Results showed that *Pichia*, *Kazachstania*, and *Saccharomyces* presented exact different effects on flavor substances (Figure 9). *Pichia* showed significantly positive correlations to ethyl acetate and main higher alcohols, whereas *Kazachstania* exhibited significantly positive relationships to lactic acid, ethyl lactate, and ethyl phenyl-lactate (Figure 9). Furthermore, *Saccharomyces* was significantly positive to the formation of partial ethyl esters, especially the ethyl esters of MCFAs (Figure 9). These positive relationships were consistent with the metabolic capacities of the three yeasts mentioned in Figure 4, further verifying the functions of these three dominant yeasts during baijiu brewing.

## 4. Discussion

Yeasts are the crucial microbes involved in baijiu brewing since they not only yield ethanol, the main ingredient of baijiu, but produce a wide range of flavor compounds [5,27,28]. In this study, three kinds of prevailed yeast types were found during the whole brewing cycle of strong-flavor baijiu, namely *S. cerevisiae*, *P. kudriavzevii*, and *K. bulderi*. Their succession during fermentation may be ascribed to their tolerance. As fermentation went on, the ethanol and lactic acid content increased, leading to the inability of *P. kudriavzevii* to survive and to the predominance of *S. cerevisiae* and *K. bulderi* (Figure 2B). Ultimately, *K. bulderi* was the only remaining species due to its excellent tolerance to lactic acid (Figure 2B). Wu’s study found that *K. exigua*, another species of genus *Kazachstania*, also showed extremely strong acid tolerance and could even survive under condition of pH 2.3 [27], presenting better acid tolerance far exceeding that of *Pichia* and *S. cerevisiae*. The applying H^+^ extrusion and glycerol transportation of *Kazachstania* to resist the stress from lactic acid may explain this [29]. Nonetheless, the amount of *K. bulderi* is very low at the end of fermentation (Figure 2B) because harsh conditions (high acidity and ethanol, poor nutrition, and strict anaerobic conditions) of fermented grains in mud-pits makes it difficult for yeast to survive and grow [27,28,30].

After the dominant yeasts were identified, the metabolic profiles of *S. cerevisiae* FJ1-2, *P. kudriavzevii* FJ1-1, and *K. bulderi* FJ1-3 were analyzed. *S. cerevisiae* FJ1-2 yielded 26.70 g/L of ethanol during static fermentation for 5 days (Figure 4), whereas *S. cerevisiae* MT1, isolated from sauce-flavor baijiu, only produced 11.12 g/L of ethanol under the condition of 200 rpm for 2 days [31]. This discrepancy was caused by the differences in strain instinct and culture condition, and yeasts isolated from different flavors of baijiu would exhibit distinct metabolic characteristics [28,32]. *S. cerevisiae* FJ1-2 also had a remarkable potential for producing MCFAs and their ethyl esters (Figure 4), which are the main aroma compounds of alcoholic beverages [33], attributing to the flavor of baijiu [2,4,6]. Further, the outstanding facility for generating ethyl acetate by *P. kudriavzevii* FJ1-1was found (Figure 4), proving that this species had strong ability in the production of ethyl acetate [34,35]. *K. bulderi* FJ1-3 had only half the ethanol output of *S. cerevisiae* FJ1-2 and *P. kudriavzevii* FJ1-1, but it gained a better aptitude for producing organic acids, particularly lactic acid and propionic acid (Figure 4), revealing the metabolic diversity among yeast species isolated from baijiu brewing [18]. Previous studies have highlighted that yeasts acclimate to the alcoholic fermentation system (wine or baijiu) to survive in a hostile environment, leading to metabolism diversity [27,28,36]. In summary, these three dominant yeasts displayed different aptitudes for producing ethanol and flavor substances, regulating the baijiu flavor by their adaptations during fermentation.

Subsequently, these three dominant yeasts were prepared as reinforced *Fuqu* independently and applied severally to verify their functions during baijiu brewing at lab scale. During fermentation, microbes consumed reducing sugar and hence produced water, ethanol, organic acids, and other flavor substances. The generated water affected moisture [18], while the resultant organic acids influenced the acidity and pH [37]. Meanwhile, the moisture, acidity (pH), and reducing sugar content of fermented grains affected the microbial compositions and succession in turn [38,39]. Concerning the fungal community during baijiu brewing, the results by amplicon sequencing at lab scale (Figure 7E) were different from those by the culturable method in situ (Figure 2). This might be caused by the different fermentation environment, specifically in the distinct anaerobic degree between plastic boxes at lab and mud-pits in situ [18]. Although this discrepancy existed, the abundance of enhanced yeast in each group was distinctly increased in contrast with CK group (Figure S3), indicating the reinforced yeast could quickly adapt to brewing environment and then capture the niche [22,24,40]. Totally, adding *S. cerevisiae* FJ1-2 or *P. kudriavzevii* FJ1-1 significantly crowded out the ecological niche of *Kazachstania*; however, reinforcing *K. bulderi* FJ1-3 occupied the partial ecological niche of *Pichia* and *Saccharomyces* (Figure 7). Wang found that reinforcing *Wickerhamomyces anomalus* would obviously decrease the abundance of *Pichia* during baijiu production, further indicating that fortification is a good means to enhance the niche of target yeast and then exert its function [40].

As for flavor substances, higher alcohols are important in baijiu, but their high concentration will bring negative effects on health, such as headaches after drinking [18,32,41]. In this situation, reinforcing *K. bulderi* FJ1-3 decreased the higher alcohols content compared with that of strengthening *S. cerevisiae* FJ1-2 or *P. kudriavzevii* FJ1-1 during baijiu brewing at lab scale application (Figure 6A). Our previous study observed that *S. cerevisiae* and *P. kudriavzevii* were the dominant microbes involved in higher alcohols formation during baijiu brewing via meta-transcriptomics analysis [41], supporting the above result. Li et al. reported a method to reduce the higher alcohol content by sequential fermentation with *S. cerevisiae* and non-*Saccharomyces* yeasts (*Zygosaccharomyces bailii* and *P. kudriavzevii*) during winemaking [42]. Furthermore, acetic acid, lactic acid, butyric acid, and caproic acid are the most decisive organic acids responsible for the favor of baijiu [18,27]. The enhancement of *K. bulderi* FJ1-3 promoted the total organic acids content (Figure 6B). Additionally, esters are the main flavor substances in baijiu, whose proportion accounts for over 60% of the total flavor substances [2,11,43]. The total esters content of these three reinforced groups was similar (Figure 6C), but the level of ethyl acetate and ethyl caproate differed in three groups (Figure 6D and Figure S5F). The ratio of ethyl caproate to ethyl acetate was higher in KF group than that in SF and PF groups (Figure S4). However, the ratio of ethyl caproate to ethyl acetate in SF should be the highest because *S. cerevisiae* FJ1-2 demonstrating the best capability for producing ethyl caproate (Figure 3J). We speculated that the reinforcement of *S. cerevisiae* FJ1-2 regulated the yeast community by promoting the abundance of *Pichia* (Figure S3) and thus increasing the level of ethyl acetate as well (Figure 6D), which might account for the above phenomenon. Meanwhile, intensifying *K. bulderi* FJ1-3 lowered the abundance of *Pichia* and promoted the abundance of *Saccharomyces* (Figure S3), thus decreasing the content of ethyl acetate and increasing the level of ethyl caproate (Figure 6D,F). Coincidentally, ethyl caproate is recognized as the main flavor compound in the national standard for strong-flavor baijiu, whose high level is significantly related to high quality [2,25]. Here, reinforcing dominant yeast could be an effective manner to better baijiu quality, since the application of dominant yeast ensures that it can quickly adapt to the brewing environment and function effectively. The yeast types, abundances, and interactions had critical impacts on baijiu fermentation, especially the flavor formation [23,25,44]. Therefore, isolating dominant yeasts from the brewing processes of strong-flavor baijiu would be the basis for studying their characteristics and functions, providing potential microbial resources for quality improvement of baijiu. Conclusively, *K. bulderi* FJ1-3 could modulate the yeast community, enhancing baijiu quality by elevating ethyl caproate level while lowering ethyl acetate and higher alcohols during strong-flavor baijiu brewing.

## 5. Conclusions

In this study, three dominant yeasts were identified from strong-flavor baijiu, namely *S. cerevisiae*, *P. kudriavzevii*, and *K. bulderi*. Among them, *S. cerevisiae* FJ1-2 displayed superior abilities in ethanol resistance, ethanol production, and the formation of ethyl esters of MCFAs. *P. kudriavzevii* FJ1-1 demonstrated the highest thermal tolerance and capacity for ethyl acetate production. Meanwhile, *K. bulderi* FJ1-3 exhibited the highest lactic acid tolerance, which likely contributes to the observed yeast successions during strong-flavor baijiu fermentation. Subsequently, these three yeasts were prepared as reinforced *Fuqu* and applied to baijiu brewing at lab scale to verify their functions, severally. The relative abundance of each fortified yeast increased in comparison with CK group. *Pichia*, *Kazachstania*, and *Saccharomyces* emerged as the core microbe for each group, respectively, by co-occurrence network analysis. The reinforced yeast in *Fuqu* exhibited the ability to regulate microbiota, thus altering the physicochemical factors and flavor substances during fermentation. In summary, fortifying *K. bulderi* FJ1-3 effectively controlled the populations of *Pichia* and *Saccharomyces* and led to the increase in ethyl caproate together with the decrease in ethyl acetate and higher alcohols during baijiu brewing, contributing to the quality improvement of strong-flavor baijiu. This is a meaningful attempt to verify the functions of yeasts by applying fortified *Fuqu*, which will provide potential yeasts resources for improving the quality of strong-flavor baijiu.

## Figures and Tables

**Table 1:** The group sets of reinforced *Fuqu* application during baijiu brewing at lab scale.

Group Sets	Parameters
CK group	20 kg brewed grains + 2.0 kg *Daqu*
SF group	20 kg brewed grains + 1.8 kg *Daqu* + 0.2 kg SF
PF group	20 kg brewed grains + 1.8 kg *Daqu* + 0.2 kg PF
KF group	20 kg brewed grains + 1.8 kg *Daqu* + 0.2 kg KF

## Data Availability

The original contributions presented in the study are included in the article and Supplementary Material, further inquiries can be directed to the corresponding author.

## Supplementary Materials

The following supporting information can be downloaded at: https://www.mdpi.com/article/10.3390/foods13152409/s1, Figure S1: The phylogenetic tree (neighbor-joining) of the isolated three dominant yeasts; Figure S2: The variations of flavor substances in fermented grains with the application of reinforced *Fuqu* during baijiu brewing at lab scale; Figure S3: The average abundance of three yeasts during baijiu brewing at lab scale with the application of reinforced *Fuqu*; Figure S4: The ratio between ethyl caproate and ethyl acetate during baijiu brewing with reinforced *Fuqu*.
