# Supplementary material for: Characteristics and Functions of Dominant Yeasts Together with Their Applications during Strong-Flavor Baijiu Brewing
Source: Foods. 2024 Jul 30;13(15):2409. doi: 10.3390/foods13152409 (PMC11311647; doi:10.3390/foods13152409)
Supplement: Supplementary file 1 [file foods-13-02409-s001.zip › foods-3060864-supplementary.docx]

**Supplementary materials for**

***Foods***

**Characteristics and functions of dominant yeasts together with their applications during strong-flavor baijiu brewing**

Weiwei Dong^1, 2^, Yulun Zeng^2^, Jiyuan Ma^1^, Kaiyun Cai^3^, Tingting Guo^3^, Guangxun Tan^3^, Xiang Yu^1^, Yuanliang Hu^1^, Nan Peng^2^, Shumiao Zhao*^2^

^1^ Hubei Key Laboratory of Edible Wild Plants Conservation and Utilization, College of Life Sciences, Hubei Normal University, Huangshi 435002, China.

^2^ National Key Laboratory of Agricultural Microbiology and College of Life Science and Technology, Huazhong Agricultural University, Wuhan 430070, China.

^3^ Hubei Daohuaxiang Liquor Co., Ltd, Yichang 443112, China.

*Corresponding authors:

Shumiao Zhao, Tel/fax: +8602787281040; E-mail address: shumiaozhao@mail.hzau.edu.cn

Method for sorghum juice medium:

Two hundred grams of sorghum was mixed with 800 mL of deionized water and steamed at 105 °C for 2 h. After cooling to 65 °C, α-amylase (5000 U/L) was added and then incubation was conducted at 65 °C for 4 h with stirring. Subsequently, saccharification was carried out by adding glucoamylase (5000 U/L) under 50 °C for 4 h. Afterward, the supernatant of the mixture was obtained by filtering through four layers of gauze and centrifuging at 8000g for 15 min. Hence, the sugar content of collected sorghum juice was diluted with water to 10 ° Bx by a Leica refractometer. At last, sorghum juice medium was sterilized at 115 °C for 30 min for further use.

Method for PCR and Sanger sequencing of yeast strains:

The single colony of related yeast strain was picked into a 1.5 mL sterile Eppendorf tube. The lysis buffer (5% Chelex-100 resin, autoclaved) was then added to the above tube. After suspension, the mixture was heated at boiling water bath for 10 min and immediately transferred to frozen condition in a refrigerator at -20 °C for 10 min, which benefits to the release of yeast DNA. Afterward, the supernatant was obtained by centrifuging the mixture at 6000 rpm for 2 min, and the supernatant was then used as template for PCR. Here, the primers, including NL1 (5’-GCATATCAATAAGCGGAGGAAAAG-3’) and NL4 (5’-GGTCCGTGTTTCAAGACGG-3’), together with Taq DNA polymerase were used for amplification, following the parameter: 95 °C for 5 min, 95 °C for 15 s, 53 °C for 15 s, and 72 °C for 10 s for 30 cycles, and final at 72 °C for 5 min. After purification, the PCR products were sequencing by Tsingke Biotech Co., Ltd. (Wuhan, China) based on Sanger sequencing. The resultant sequence of related single colony was BLAST in NCBI (https://blast.ncbi.nlm.nih.gov) and Yeasts (https://theyeasts.org/page/Homepage) for searching the close sequences with their species names. Finally, all these close sequences together with the target sequence were used to construct phylogenetic tree to identify the taxonomic status of yeast.

*Saccharomyces bayanus* NRRL Y-12624

*Saccharomyces pastorianus* NRRL Y-27171

*Saccharomyces kudriavzevii* ATCC MYA-4449

*Saccharomyces arboricola* CBS 10644

*Saccharomyces mikatae* ATCC MYA-4448

*Saccharomyces cariocanus* NRRL Y-27337

*Saccharomyces paradoxus* NRRL Y-17217

*Saccharomyces cerevisiae* UMCC 2988

*Saccharomyces cerevisiae* CLIB 3067

***Saccharomyces cerevisiae* FJ1-2**

*Saccharomyces cerevisiae* NRRL Y-12632

*Kazachstania pseudohumilis* CBS 11404

*Kazachstania humilis* NRRL Y-17074

*Kazachstania exigua* CBS 379

*Kazachstania saulgeensis* CLIB 1764

*Kazachstania turicensis* CBS 8665

***Kazachstania bulderi* FJ1-3**

*Kazachstania bulderi* 30-1

*Kazachstania bulderi* PD-1

*Kazachstania bulderi* KKKS4-1

*Kazachstania bulderi* MUCL 38021

*Pichia occidentalis* NRRL Y-7552

*Pichia exigua* NRRL Y-10920

*Pichia scutulata* NRRL Y-7663

*Pichia kudriavzevii* Y6

*Pichia kudriavzevii* 4Y211

*Pichia kudriavzevii* DGY49

***Pichia kudriavzevii* FJ1-1**

*Pichia kudriavzevii* CK9

26

43

44

100

77

79

57

33

31

88

84

61

97

79

98

100

98

78

69

96

95

32

23

43

17

34

0.050

Figure S1 The phylogenetic tree (neighbor-joining) of the isolated three dominant yeasts (labeled in red, numbers at the nodes are the bootstrap values from 1,000 replicates). We blast the sequence in the database of NCBI (https://blast.ncbi.nlm.nih.gov) and Yeasts (https://theyeasts.org/page/Homepage) and accessed data in Sep 2022.





Figure S2 The variations of flavor substances in fermented grains with the application of reinforced Fuqu during baijiu brewing at lab scale, including ethanol (A), isoamylol (B), phenethyl alcohol (C), acetic acid (D), lactic acid (E), caproic acid (F), ethyl acetate (G), ethyl lactate (H), ethyl butyrate (I), ethyl caproate (J), ethyl phenylacetate (K), and ethyl phenylpropionate (L).


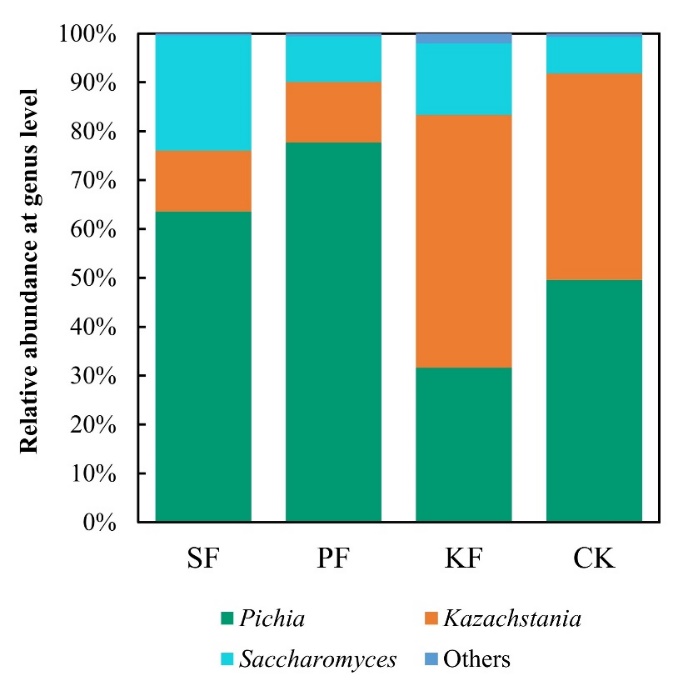


Figure S3 The average abundance of three yeasts during baijiu brewing at lab scale with the application of reinforced *Fuqu*.


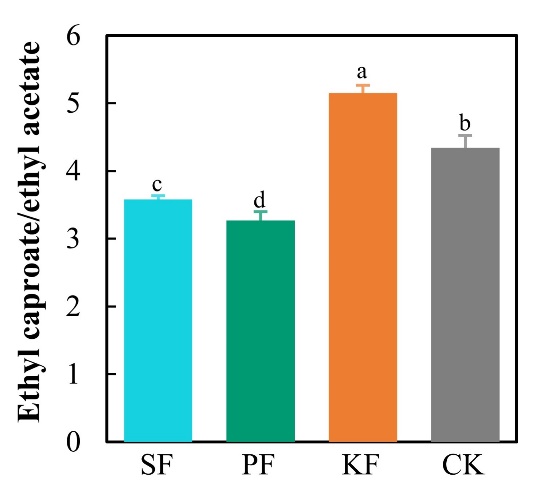


Figure S4 The ratio between ethyl caproate and ethyl acetate during baijiu brewing with reinforced *Fuqu*. The differences were evaluated by significant at p<0.05 with the software Origin (version 9.0) via One-Way ANOVA test.
